# Elevated Peptides in Lung Lavage Fluid Associated with Bronchiolitis Obliterans Syndrome

**DOI:** 10.1371/journal.pone.0084471

**Published:** 2014-01-02

**Authors:** Matthew D. Stone, Stephen B. Harvey, Gary L. Nelsestuen, Cavan Reilly, Marshall I. Hertz, Chris H. Wendt

**Affiliations:** 1 Waters Corporation, Milford, Massachusetts, United States of America; 2 Department of Biochemistry, Molecular Biology and Biophysics, University of Minnesota, Minneapolis, Minnesota, United States of America; 3 Department of Biostatistics, University of Minnesota, Minneapolis, Minnesota, United States of America; 4 Department of Medicine, University of Minnesota, Minneapolis, Minnesota, United States of America; 5 Department of Medicine, Veterans Administration Medical Center, University of Minnesota, Minneapolis, Minnesota, United States; University of California Los Angeles, United States of America

## Abstract

**Objective:**

The objective of this discovery-level investigation was to use mass spectrometry to identify low mass compounds in bronchoalveolar lavage fluid from lung transplant recipients that associate with bronchiolitis obliterans syndrome.

**Experimental Design:**

Bronchoalveolar lavage fluid samples from lung transplant recipients were evaluated for small molecules using ESI-TOF mass spectrometry and correlated to the development of bronchiolitis obliterans syndrome. Peptides associated with samples from persons with bronchiolitis obliterans syndrome and controls were identified separately by MS/MS analysis.

**Results:**

The average bronchoalveolar lavage fluid MS spectrum profile of individuals that developed bronchiolitis obliterans syndrome differed greatly compared to controls. Controls demonstrated close inter-sample correlation (R = 0.97+/−0.02, average+/−SD) while bronchiolitis obliterans syndrome showed greater heterogeneity (R = 0.86+/−0.09, average+/−SD). We identified 89 features that were predictive of developing BOS grade 1 and 66 features predictive of developing BOS grade 2 or higher. Fractions from MS analysis were pooled and evaluated for peptide content. Nearly 10-fold more peptides were found in bronchiolitis obliterans syndrome relative to controls. C-terminal residues suggested trypsin-like specificity among controls compared to elastase-type enzymes among those with bronchiolitis obliterans syndrome.

**Conclusions:**

Bronchoalveolar lavage fluid from individuals with bronchiolitis obliterans syndrome has an increase in low mass components detected by mass spectrometry. Many of these features were peptides that likely result from elevated neutrophil elastase activity.

## Introduction

Lung transplantation has become a widely accepted therapeutic modality for many end-stage lung diseases. Unfortunately, chronic rejection remains a major barrier to long-term survival with 50–60% of lung transplant recipients affected at 5 years [1], [2]. The clinical surrogate of chronic allograft rejection is bronchiolitis obliterans syndrome (BOS), which manifests as a decline, often progressive, of lung function [3]. In addition to a peribronchial infiltration of lymphocytes, neutrophilia also is a predominant finding in BOS [4]–[9]. These neutrophils release factors of the innate immune system, along with proteases [4], [10]–[12]. Despite the high incidence of BOS, the underlying pathogenesis remains unknown and no clinical biomarker has been found to predict its onset.

Bronchoalveolar lavage fluid (BALF) is a rich source of potential biomarkers. Although invasive, BALF is collected on a routine basis for surveillance after lung transplant. In addition to cells and proteins, small molecules or metabolites are sampled in BALF. These small molecules can represent the end product of metabolism, lipids, drugs and peptide byproducts of protease digestion. Identification of metabolites unique to BOS has the potential to provide novel biomarkers and to provide insight into mechanism of disease. This report describes a discovery-level study of BALF samples from individuals at various times before and after the diagnosis of BOS. While specific metabolites were targeted, it became clear that many were peptides generated by protease activity.

## Materials and Methods

### Study Population

The samples studied were excess cell-free BALF collected between 1993–1996 and 2003–2007 at the time of clinically obtained bronchoscopies as previously described [10]. Written informed consent was obtained from all subjects. Briefly, bronchoalveolar lavage consisted of 100–140 ml normal saline instilled in either the right middle lobe or left upper lobe (lingula), then aspirated. Samples were collected and immediately placed on ice, cells were removed by centrifugation and supernatants were stored at –80°C [10]. For this case-control study we chose 145 BALF samples (summarized in Table 1) from 64 individuals ages 27–63 (median 49, 48% female, average 2.25 BALF samples/individual, range 1–8). Of these samples, 76 were from the 1993–1996 source described previously [10] and 69 were from the 2003–2007 source. The sample size was determined by the number that could be accommodated in a single MS run. Cases (n = 95) consisted of individuals that developed BOS within 18 months of BALF sample acquisition. Controls (n = 50) were defined as those who did not progress to any grade of BOS within 6 years or more from BALF collection. Due to differences in column properties and elution times, LC-MS data from the two experiments could not be combined, whereas peptide and protein identifications were combined.

**Table 1:** Subject underlying diseaseunderlying Disease.

Underlying Disease	Controls (n = 50)	BOS (n = 95)
COPD/Emphysema	19 (38.0%)	38 (40%)
Alpha -1- Antitrypsin Deficiency	13 (26.0%)	37 (38.9%)
Pulmonary Hypertension	3 (6.0%)	12 (12.2%)
Cystic Fibrosis/Bronchiectasis	9 (18.0%)	0 (0%)
Pulmonary Fibrosis	4 (8.0%)	8 (8.4%)
Other	2 (4.0%)	0 (0%)

### Ethics Statement

This study was approved by the University of Minnesota Institutional Review Board Human Subjects Committee (# 0107M04822).

### Sample Preparation and Initial LC-MS Analysis

BALF samples (0.80 mL) were prepared for LCT-MS analysis by application to a disposable C18 spin column (MacroSpin, C18, The Nest Group Inc.). Columns were conditioned with 500 µL acetonitrile (ACN) followed by 500 µL water with centrifugation (4 minutes, 2000×g) each time. Samples were acidified to pH 2 with formic acid and applied to the conditioned column. The columns were rinsed twice with 400 µL of water/ACN/formic acid (95/5/0.1). The sample was eluted first with 200 µL of water/ACN/formic acid (50/50/0.1) followed by 200 µL of water/ACN/formic acid (10/90/0.1). The combined 400 µL was concentrated by vacuum centrifugation to approximately 50 µL. Samples were brought up to 100 µL with water/ACN/formic acid (95/5/0/1). Analysis was performed with the Waters, Inc. Acquity UPLC system and an Acquity UPLC BEH C18 1.7 µm 2.1×100 mm column. The flow rate was 0.5 mL/minute with a column temperature of 40°C. Injected sample (7 µL) was eluted using an increasing gradient of ACN/water in 0.1% formic acid: 0.5 minutes of initial buffer (water:can,98∶2), a gradient to 20% ACN (over 0.5 minutes) and a linear gradient to 100% ACN (over 7 minutes). The column was washed with 100% ACN (two minutes) and then re-equilibrated with the starting buffer. This protocol provided acceptable peak separation with minimal carry-over. Mass spectrometry utilized the Waters LCT Premier XE TOF operated in positive, double reflectron mode with dynamic range enhancement. The scan rate was 0.1/s with continual calibration by a lock spray. Leucine enkaphalin was the lock spray reagent (557.2802 lock mass and 556.2771 attenuated lock mass) sampled every ten scans. Capillary and cone voltages were 2500 and 30 V, respectively. Features (accurate mass and retention time pair) of the profile were compiled using MarkerLynx software and were expressed as either raw intensity or normalized to 10,000 total counts per profile. Features of the background (>0.1-times the sample average) were disregarded. A feature is defined by a specific mass/charge (m/z) and retention time. Due to multiple charge states and possible adducts, a metabolite may be represented by more than one feature.

### Peptide Identification by LC-MS/MS

Peptide identification required pooling of samples from the LCT-MS run to reach adequate concentrations for MS/MS analysis. In two separate experiments we pooled aliquots (20 µL each) from: 1) 6 BOS samples and from 16 control samples (1993–96 samples) and 2) 15 controls and 5 BOS samples (2003–2007 samples). UPLC (elution as above) fractions were collected at 0.5 minute intervals (0.25 mL) between 1.0 and 3.5 minutes. Each fraction was submitted for MS/MS analysis in the Orbitaltrap mass spectrometer for peptide identification. Sample loading, HPLC, and mass spectrometry were performed as previously described [13] with the following exceptions: Electrospray mass spectrometry was performed using a LTQ-Orbitrap XL (ThermoScientific) with spray voltage set to 1.95 kV. Charge state screening was enabled so that undetermined charge states were excluded for data dependent fragmentation. Each sample was run in duplicate to enhance coverage.

### Database Searching and Data Processing

Raw mass spectrometric data obtained from Xcalibur software (ThermoScientific) were extracted using ReAdw (Institute of Systems Biology) to generate mzXML files. Data were searched with SEQUEST V27 against a composite database consisting of the NCBI human database V200806 and its reversed complement and common contaminating protein sequences totaling 70711 entities. A total of 42242 spectra were searched from the control dataset and 65124 spectra were searched from the BOS dataset. Search parameters included no enzyme specification, 100 ppm precursor molecular mass tolerance, 0.8 amu fragment ion mass tolerance, a precursor ion mass range from 700–3600 Da and Met oxidation. SEQUEST output was organized and peptide probabilities were calculated through Peptide Prophet [14] using Scaffold (Proteome Software, Inc., Portland, OR). Peptide identifications were filtered using the following parameters: 95% peptide probability and 7 ppm for precursor mass tolerance. Estimated false positive rates were calculated from identified spectra using the equation: (2 × reverse database identifications/(forward+reverse database identifications)*100). Protein false discovery rates were determined using the following calculation: (reverse database identifications/(forward+reverse database identifications)*100). All identified MS/MS spectra (Table S1) can be downloaded from Tranche (https://proteomecommons.org/tranche/) with the following hash: haakgsT9g6Fl4waTlcLGNCD7NJepCE2t0YG6oZCvq6dvkBc7uwf25BFEtTZ9hff0szXET8NKsODQcM3SeC0gkdaLGoUAAAAAAAACbA =  = . These annotated spectra can be viewed using the freely available Scaffold Viewer software from www.proteomesoftware.com.

### Statistical Analysis

Regression analysis was used to obtain correlation coefficients for comparison of total LCT-MS profiles. To test if a metabolomic feature was predictive of time to development of BOS, the median intensity of each feature was used to dichotomize the feature values. The log rank test was used to test for a difference in the time to development of BOS between the dichotomized feature values. The p-values produced by the log rank test were then adjusted for multiple comparisons using the q-value approach of Storey [15]. Features were declared of interest by setting the threshold for the false discovery rate at 10%. This analysis was conducted separately for BOS grade 1 and BOS grade 2 or 3.

## Results

### LCT-MS Analysis

**Figure 1:**
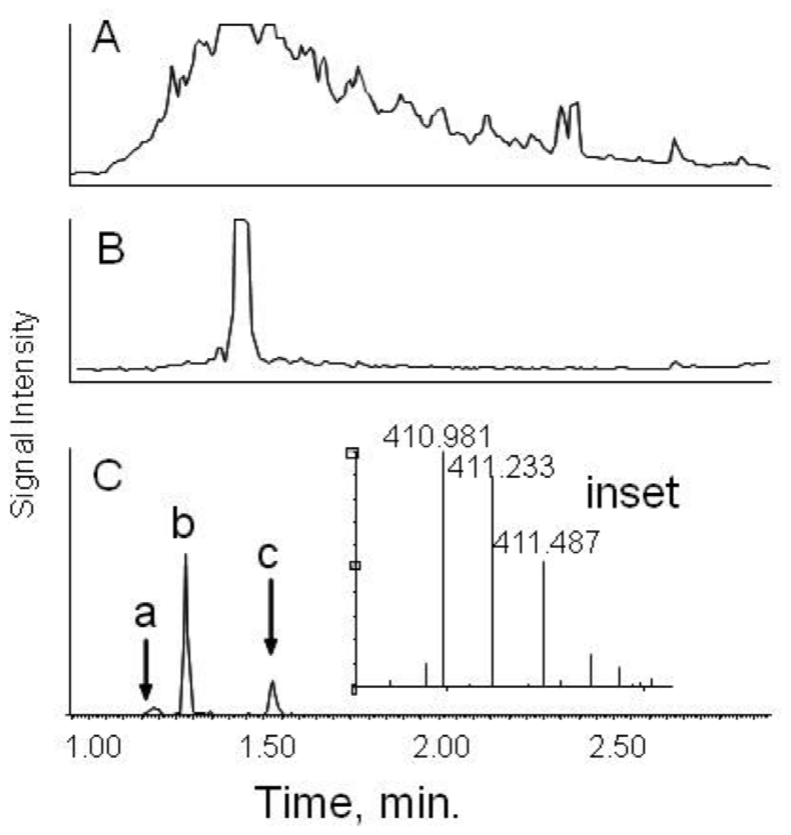
UPLC separation of BALF fluid. Panel A. Total ion current for a typical sample from an individual with BOS. Panel B. Total ion current for a control subject who did not develop BOS within at least 100 months. The intensity has been adjusted to the same maximum as Panel A. The large peak at 1.48 minutes corresponded to lidocaine. Panel C. Extracted ion current for the +4 charge state of a component at m/z = 410.98. Only peak b represented a monoisotopic ion. Peaks a and c were isotopes of other compounds.

The total ion current from LCT-MS analysis of organic solvent-soluble materials revealed a very intense peak at 1.48 minutes with m/z = 235.34. This was consistent with lidocaine, the local anesthetic used in the bronchoscopy. Excluding that feature, profiles were widely divergent between controls and those with BOS. An individual with BOS (Figure 1A) showed very high total ion current between 1.2 to 3.5 minutes while many control samples showed low intensity for most of the profile (example in Figure 1B).

From visual observation it was apparent that the average profile for BOS differed greatly from the average profile for controls. To determine reproducibility, replicate experimental runs were performed at a 24-hour interval on 6 BOS samples and 6 controls. Reproducibility was excellent as illustrated by high correlation coefficients of 0.996–0.999 between experimental runs. Inter-sample comparisons gave high correlation coefficients as well, especially for controls (R = 0.97+/−0.02). All combinations among six BOS samples showed somewhat lower correlation (R = 0.86+/−0.09). Most surprising was that BOS samples had similar correlation to control samples (R = 0.86+/−0.07). Overall, correlation coefficients indicated that the novel features of BOS samples differed widely, whereas control samples contained similar features.

**Figure 2:**
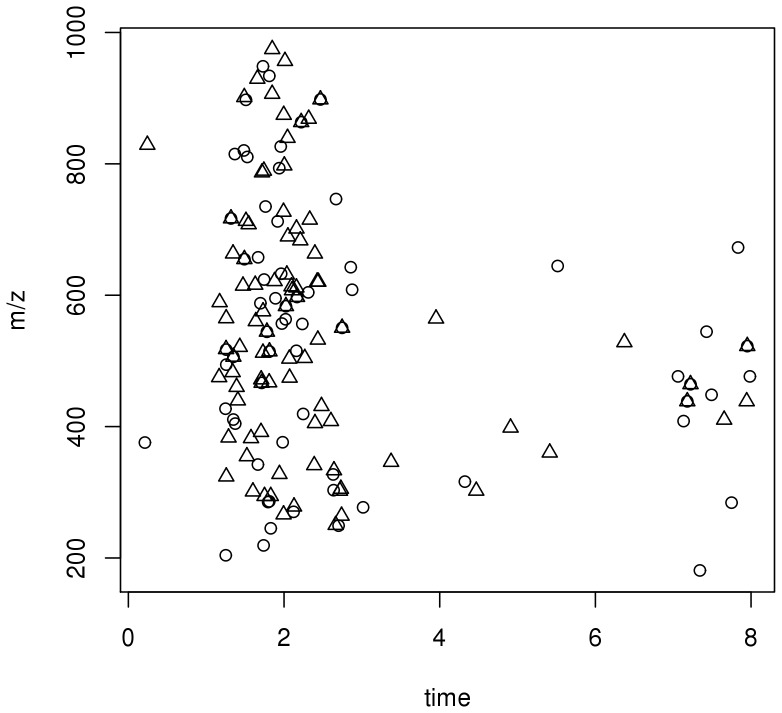
Features predictive of BOS. The median intensity of each feature is represented by the mass/charge (m/z) and retention time (time). Using a false discovery rate of 10%, the circles indicate features that are predictive of time to BOS 1 (triangles, n = 89) and those that are predictive of time to BOS 2 or 3 (circles, n = 66).

To test if a feature was predictive of developing BOS we used the log rank test to look for a difference in the time to develop BOS. Since we had multiple samples from individuals (average 2.25 BALF samples/individual) we used the first BALF sample obtained from an individual for analysis. We identified 89 features that were predictive of developing BOS grade 1 and 66 features predictive of developing BOS grade 2 or higher (Figure 2). Most of these features had elution times, mass ranges (m/z) and charge states that were suggestive of peptides.

One limitation of the LCT-MS is that it provided m/z and retention times but not structural identities. Circumstantial factors such as elution times and the presence of multiply charged features suggested that many of the features predictive of BOS were peptides. An example was a feature at +4 charge that eluted at 1.33 minutes (peak b, Figure 1C). A peptide of the same m/z and charge state was found in separate MS/MS analysis of pooled samples and was assigned IRNDEELNKLLGKV of Histone cluster 1, H2ah (Table S2). The extracted ion profile showed two minor peaks with the same m/z (Figure 1C, lower case a and c). However, these were easily distinguished as isotopes of other compounds. We sought to identify endogenous peptides in our samples since the retention times and charge pattern of many of the features that predicted BOS suggested that they were peptides.

### Identification of Endogenous Peptides of BALF

To have adequate sample volume we used pooled samples from the BOS and control sample UPLC chromatograms to identify endogenous peptides. Fractions were collected from two column runs from each sample source and were analyzed in the LTQ-Orbitrap mass spectrometer. Peptide identifications were stringently filtered to give an estimated false positive rate of 0.16%, corresponding to a protein false discovery rate of 1.2%. A comprehensive list of all unique peptide identifications is given in Table S2. Controls provided 196 unique peptides from 349 assigned spectra. From these peptides we identified 45 proteins. The BOS samples had 1739 unique peptides from 3424 identified spectra and a total of 228 unique proteins identified from the peptides (Table S3). The majority (84%) of the 45 proteins identified in controls were also found in BOS. There was less commonality among peptides; only 34% of the peptides found in the control samples were identified in BOS. Therefore, although there appears to be some common substrates (i.e. proteins) that generate the various peptides, the peptides themselves are heterogeneous.

**Figure 3:**
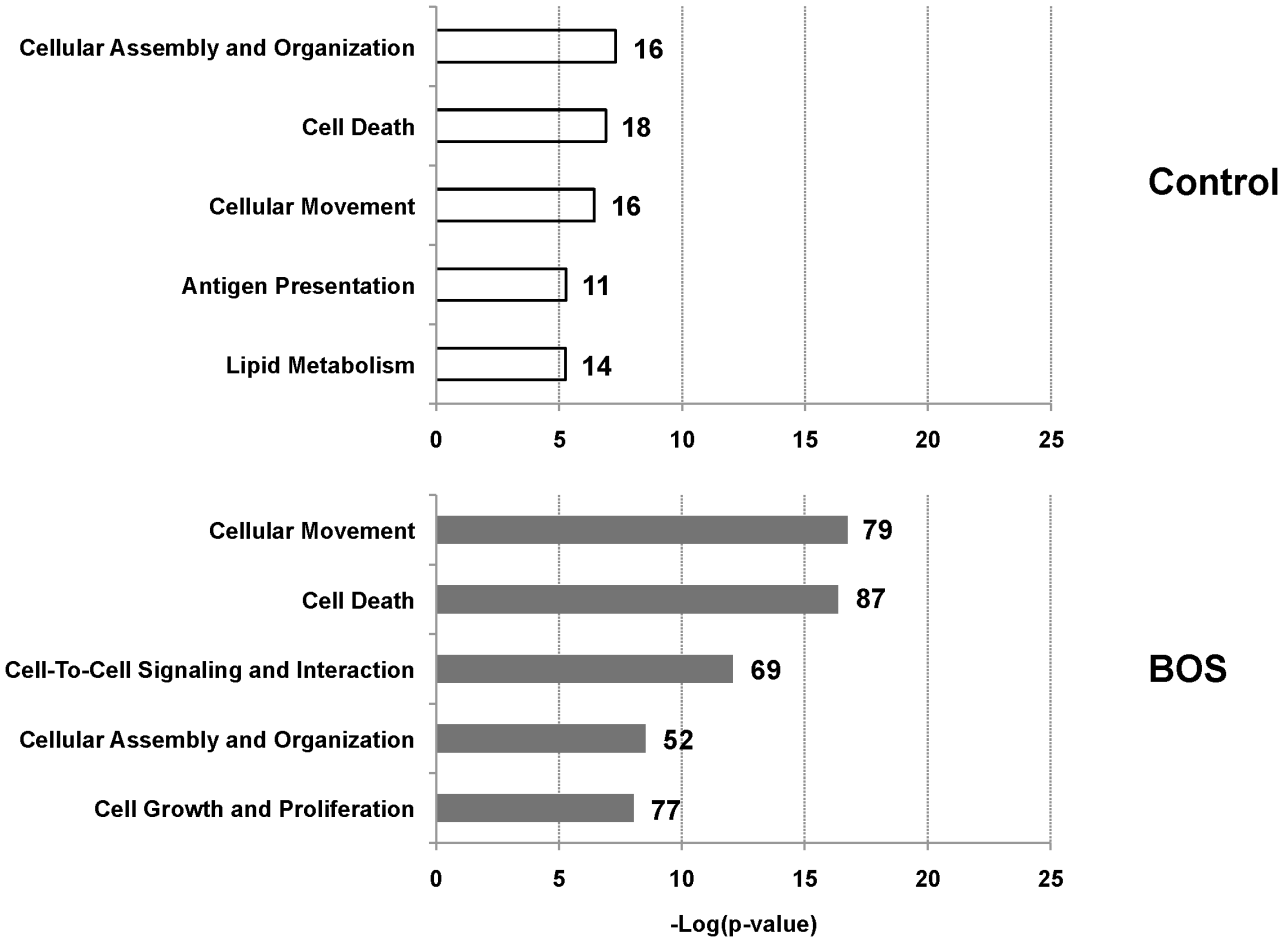
Top molecular and cellular biofunctions identified from protein substrates. Proteins identified from the sequenced peptides were used in Ingenuity Pathway Analysis to determine the molecular and cellular function of the substrate proteins. The number of proteins associated with each function is listed next to the bar.

To categorize the protein substrates that generated the peptides, Ingenuity Pathways Analysis™ (IPA) was performed using unique accession numbers of each identified peptide. The top biofunctions that the proteins identified in the BOS samples were: cellular movement, cell death, cell-to-cell signaling and interaction, cellular assembly and organization and cell growth and proliferation (Figure 3). There was overlap in biofunctions between BOS and control samples, however, there were significantly less proteins in the control samples.

Analysis of the C-terminal residues of the identified peptide sequences showed further differences between controls and BOS. C-terminals were tabulated and normalized to the natural amino acid distribution of the human genome (Table 2). Peptides from the control samples had the highest occurrence of basic C-terminal residues: 10.1 and 11.6% for K and R, respectively. BOS samples had comparatively high C-terminal frequencies of V (16.0%), I (12.5%), A (7.1%), and T (6.2%). The latter cleavage site specificity implicated an increase of elastase 2-like activity [16]. Furthermore, a peptide from elastase- 2 or human neutrophil elastase was identified in the BOS sample.

**Table 2:** C-Terminal amino acid comparison of unique endogenous peptides identified in BALF of transplant patients, control compared to BOS.

c-terminal residue	frequency	% frequency	% of frequency normalized to naturally occuring AA frequency	frequency	% frequency	% of frequency normalized to naturally occuring AA frequency
	Controls			BOS		
A	22	6.1	4.5	321	**9.4**	**7.1**
C	1	0.3	0.7	6	0.2	0.4
D	11	3.0	3.5	118	3.5	4.1
E	12	3.3	2.6	132	3.9	3.1
F	21	5.8	7.7	209	6.1	8.3
G	13	3.6	2.8	128	3.8	3.0
H	4	1.1	2.2	14	0.4	0.8
I	6	1.7	1.8	385	**11.3**	**12.5**
K	38	10.5	10.1	187	5.5	5.4
L	66	18.3	9.0	420	12.3	6.2
M	12	3.3	6.9	111	3.3	6.9
N	9	2.5	3.5	36	1.1	1.5
P	2	0.6	0.5	19	0.6	0.5
Q	19	5.3	6.0	74	2.2	2.5
R	43	11.9	11.6	86	2.5	2.5
S	33	9.1	5.7	141	4.1	2.6
T	8	2.2	2.0	232	**6.8**	**6.2**
V	14	3.9	3.4	617	**18.1**	**16.0**
W	5	1.4	4.9	21	0.6	2.2
Y	22	6.1	10.8	154	4.5	8.1
total	361			3411		

## Discussion

One initial goal of this study was to further characterize BALF of lung transplant recipients and to identify potential biomarkers for BOS. Metabolite biomarkers offer several advantages over proteins that are subject to degradation, modification and aggregation, which may confound antibody-based assays. In contrast, many small molecules are stable and easily measured. As this study progressed, it became apparent that many features of the metabolite profile were peptides. These offered the advantages of easy identification by MS/MS. The combination of peptide identity and substrate identification provides important information regarding the basis and mechanism of these peptides in BOS.

Global MS analysis revealed many components that were more abundant in patients with BOS. There was high inter-sample correlation among controls suggesting relatively uniform components of the BALF. However, profiles from BOS showed lower correlation whether compared with controls or with other BOS samples. This suggested that, while considerable similarity existed among all BALF samples, BOS samples were substantially diverse.

MS/MS analysis of pooled samples identified many peptides and their protein substrates. Ingenuity pathway analysis revealed that, while protein substrates were similar for BOS and controls, BOS samples provided 10-fold more peptides. Cellular movement, cell death and cell growth and proliferation were among the top five biofunctions of the substrates seen in BOS. This was consistent with BOS being a disease marked by loss of normal cellular function with subsequent proliferation, specifically fibroproliferation. The protein substrates present in BALF were consistent with those proteins likely to be involved in the pathogenesis of BOS.

Although we, and others, have identified the presence of certain proteases, such as matrix metalloprotease 9 and proteinase 3 as biomarkers for the diagnosis and prediction of BOS [17]–[19], specific protease activity has not been documented. Interestingly, examination of C-terminal residues indicated an elastase-2 type activity in BOS. In fact, a peptide from human neutrophil elastase was found in pooled BOS samples. Neutrophilia has been associated with BOS [4]–[6], [11], [12] and generalized protease activity has been noted to increase in BOS along with a decrease in anti-protease activity [4], [12]. Our findings suggest that although other proteases are present, the neutrophil elastase is the predominant active protease.

Overall, there were an enormous number of features in the BALF, especially in the BOS samples. Many of these features were identified as peptide metabolites generated by cleavage from neutrophil elastase. No peptide pattern specific to BOS was identified. It is possible that many other classes of small molecules are also altered in BOS and were not identified in this study. Further studies of low mass compounds may expand the number of metabolites that can be linked to the development of BOS.

## Supporting Information

Table S1 **All identified MS/MS features identified by retention time and m/z.**
(XLSX)

Table S2 **Summary of Unique Identified Peptides from Control Samples (2–199) and BOS2 Samples (202–1940).**
(XLSX)

Table S3 **Peptides matched to LCT mass spectrometry experiment.**
(XLSX)

## References

[1] Estenne M, Hertz MI (2002) Bronchiolitis obliterans after human lung transplantation. Am J Respir Crit Care Med 166: 440–444. 12186817 10.1164/rccm.200201-003pp

[2] Boehler A, Kesten S, Weder W, Speich R (1998) Bronchiolitis obliterans after lung transplantation: a review. Chest 114: 1411–1426. 9824023 10.1378/chest.114.5.1411

[3] Estenne M, Maurer JR, Boehler A, Egan JJ, Frost A, et al (2002) Bronchiolitis obliterans syndrome 2001: an update of the diagnostic criteria. J Heart Lung Transplant 21: 297–310. 11897517 10.1016/s1053-2498(02)00398-4

[4] Meyer KC, Nunley DR, Dauber JH, Iacono AT, Keenan RJ, et al (2001) Neutrophils, Unopposed Neutrophil Elastase, and Alpha(1)-Antiprotease Defenses Following Human Lung Transplantation. Am J Respir Crit Care Med 164: 97–102. 11435246 10.1164/ajrccm.164.1.2006096

[5] DiGiovine B, Lynch JP, Martinez FJ, Flint A, Whyte RI, et al (1996) Bronchoalveolar lavage neutrophilia is associated with obliterative bronchiolitis after lung transplantation: role of IL-8. J Immunol 157(9): 4194–4202. 8892657

[6] Henke JA, Golden JA, Yelin EH, Keith FA, Blanc PD (1999) Persistent increases of BAL neutrophils as a predictor of mortality following lung transplant. Chest 115: 403–409. 10027439 10.1378/chest.115.2.403

[7] Riise GC, Andersson BA, Kjellstrom C, Martensson G, Nilsson FN, et al (1999) Persistent high BAL fluid granulocyte activation marker levels as early indicators of bronchiolitis obliterans after lung transplant. Eur Respir J 14: 1123–1130. 10596701 10.1183/09031936.99.14511239

[8] Riise GC, Williams A, Kjellstrom C, Schersten H, Andersson BA, et al (1998) Bronchiolitis obliterans syndrome in lung transplant recipients is associated with increased neutrophil and decreased antioxidant status in the lung. Eur Respir J 12: 82–88. 9701419 10.1183/09031936.98.12010082

[9] Zheng L, Walters EH, Ward C, Wang N, Orsida B, et al (2000) Airway neutrophilia in stable and bronchiolitis obliterans syndrome patients following lung transplantation. Thorax 55: 53–59. 10607802 10.1136/thorax.55.1.53 PMC1745588

[10] Nelsestuen GL, Martinez MB, Hertz MI, Savik K, Wendt CH (2005) Proteomic identification of human neutrophil alpha-defensins in chronic lung allograft rejection. Proteomics 5: 1705–1713. 15800973 10.1002/pmic.200401036

[11] Elssner A, Vogelmeier C (2001) The role of neutrophils in the pathogenesis of obliterative bronchiolitis after lung transplantation. Transpl Infect Dis 3: 168–176. 11493399 10.1034/j.1399-3062.2001.003003168.x

[12] Nunley D, Dauber J, Iacono A, Keenan R, Zeevi A, et al (1999) Unopposed neutrophil elastase in bronchoalveolar lavage from transplant recipients with cystic fibrosis. American Journal of Respiratory & Critical Care Medicine 159: 258–261. 9872847 10.1164/ajrccm.159.1.9712068

[13] Bandhakavi S, Stone MD, Onsongo G, Van Riper SK, Griffin TJ (2009) A dynamic range compression and three-dimensional peptide fractionation analysis platform expands proteome coverage and the diagnostic potential of whole saliva. J Proteome Res 8: 5590–5600. 19813771 10.1021/pr900675w PMC2789208

[14] Keller A, Nesvizhskii AI, Kolker E, Aebersold R (2002) Empirical statistical model to estimate the accuracy of peptide identifications made by MS/MS and database search. Anal Chem 74: 5383–5392. 12403597 10.1021/ac025747h

[15] Storey JD (2002) A direct approach to false discovery rates. Journal of the Royal Society Statistical Society B: 479–498.

[16] Rawlings ND, Barrett AJ, Bateman A (2010) MEROPS: the peptidase database. Nucleic Acids Re 38: D227–D233. 10.1093/nar/gkp971 PMC2808883 19892822

[17] Zhang Y, Wroblewski M, Hertz MI, Wendt CH, Cervenka TM, et al.. (2005) Analysis of chronic lung transplant rejection by MALDI-TOF profiles of bronchoalveolar lavage fluid. Proteomis in press. 10.1002/pmic.200500105 16400684

[18] Hubner RH, Meffert S, Mundt U, Bottcher H, Freitag S, et al (2005) Matrix metalloproteinase-9 in bronchiolitis obliterans syndrome after lung transplantation. Eur Respir J 25: 494–501. 15738294 10.1183/09031936.05.00091804

[19] Smith GN Jr, Mickler EA, Payne KK, Lee J, Duncan M, et al (2007) Lung transplant metalloproteinase levels are elevated prior to bronchiolitis obliterans syndrome. Am J Transplant 7: 1856–1861. 17524078 10.1111/j.1600-6143.2007.01850.x

